# Supplementary material for: A broad mutational target explains a fast rate of phenotypic evolution
Source: eLife. 2020 Aug 27;9:e54928. doi: 10.7554/eLife.54928 (PMC7556874; doi:10.7554/eLife.54928)
Supplement: Supplementary file 5. [file elife-54928-supp5.docx]

***Cel-gcn-1***

**N2**: exon 21; MAL516 deletion

tcgataattttcccatttcgagacaattttcggctccaaatatgcaaaaaaaatgctccaaaattgaattttgaccaaaaattccgaaaatttaaatttaatttaaagtttttggcgtcaaattaattttttatgtttaaaatttacgaaaatgagaattttacgtgaaaagtacagtttttgagcattttttcgccgaaattcataaaaaaggataatttcggctccaaatatgcaaaaattcacatttttcccccaaaaattcacgaaaattgcatttgttttggatttttaatgcacaagaattgaattttggccgaaatttcctgaattatgcaattttttctcaaaaattcaagaaaatgagcattttttcgccaaaattcgcaaaaatatcgggaaatttaatttaaaatgctccaaaatgagcgttttttttccaatttttcccaaaaattaatctctaaaaaattccagGTAATCGTCCGAGCTCTCGGACAAAAAGATCGTGATCGTGTGCTCGCCGGGCTCTACCTGACTCGTTCCGATGTTGCTCTTGTCGTACGTCAAGCCGCTGGACACGTCTGGAAAATGGTTGTATCAAATACCCCAAGAACTCTACGAGAAGTCACGAAAATTCTCTTCGAAATGGTTGTAGACTCATTGGCTTCAACTTGTGATGAACGACAACAAATGGGTGCAAGATGTCTTGGAGAACTTGTTCGAAAAATGGGTGATAAAGTGATTAATGATATTCTACCAGTGTTGGATGCTAATCAGAAATCGGAAGAAGTCGCGAAACGCGTGGGCGTTGCTATTGCTCTTCACGAGATTATTGGAAATATGAGCAAAGAAGTCACGAATCACTATTTGGGAGCAATTGTGGCGCCGGTTAGGCGGGCGATTTGTGATGAGTCGGAACTTGTTCGTGAAGCTGCTGCTGATACGTTTACAGgtgcttgacactgcggatttttggagaaaaaagcgagaaaatcgcatttttggagaaaaaagcgagaaaatcgcatttttcgagtggaaaaatgctgaattttgtgtatttttggccaaaattcaattatttgagcatttttagcatatttgaagccgaaaattcccacctttttttttaaattttggcaaaaaaacaatttttgagtggaaaaattctcattttccagaatttttgaggaaaattgcaaaattctgcattttttggccaaaattcaattatttgagcatttttagcatatttgaagccgaaaattcccacctttttttttaaattttggcaaaaaaacaatttttgagtggaaaaattctcattttccagaatttttgaggaaaattgcaaaattctgcattttttggccaaaattcaatttttgagcatttttagcatatttggagccgtaaattgtcccttttttgtgaatttttgagaaaaaatcaaattttgagcattttttgagacaaaaattggaaaaattccgatttttttgcgatttttcaagtagaaaaatgctcatttttgtgaattttaggttcacattgcaaatttatgcattttttagccaaaattcgaattttgagcattttaaacatatttggagctaaaaattgtcccttttttgtgaatttttggcgaaaaatgctcagaattgcagtttttatgtaaaattctcattgtcgtaattttacgcatgaaaaaatgaattttaggttgaaaat

**MAL516**: deletion breakpoint

tcgataattttcccatttcgagacaattttcggctccaaatatgcaaaaaaaatgctccaaaattgaattttgaccaaaaattccgaaaatttaaatttaatttaaagtttttggcgtcaaattaattttttatgtttaaaatttacgaaaatgagaattttacgtgaaaagtacagtttttgagcattttttcgccaaaattcgaattttgagcattttaaacatatttggagctaaaaattgtcccttttttgtgaatttttggcgaaaaatgctcagaattgcagtttttatgtaaaattctcattgtcgtaattttacgcatgaaaaaatgaattttaggttgaaaat

**JU3641**[*gcn-1(mf165)*]: deletion breakpoint; intronic SNPs

tcgataattttcccatttcgagacaattttcggctccaaatatgcaaaaaaaatgctccaaaattgaattttgaccaaaaattccgaaaatttaaatttaatttaaagtttttggcgtcaaattaattttttatgtttaaaatttacgaaaatgagaattttacgtgaaaagtacagtttttgagcattttttcgccacaactctaacttcgaacacttcaaccacatttagagctaaaaattgtcccttttttgtgaatttttggcgaaaaatgctcagaattgcagtttttatgtaaaattctcattgtcgtaattttacgcatgaaaaaatgaattttaggt

**JU3642**[*gcn-1(mf166)*]: deletion breakpoint; intronic SNPs tcgataattttcccatttcgagacaattttcggctccaaatatgcaaaaaaaatgctccaaaattgaattttgaccaaaaattccgaaaatttaaatttaatttaaagtttttggcgtcaaattaattttttatgtttaaaatttacgaaaatgagaattttacgtgaaaagtacagttttttaacattttttcggagctaaaaattgtcccttttttgtgaatttttggcgaaaaatgctcagaattgcagtttttatgtaaaattctcattgtcgtaattttacgcatgaaaaaatgaattttaggttgaaaat

***Cel-cdk-8***

**PB306**: exon 2; MAL450 SNP T>C (V40A)

ttggaaatttgcactataaataaatcagaaaatccctattgcagATTAATGATTGATGAAAACTTCAAAAAACAATTGGCACAGCGTCGAGAGAGAGTAGAAGACCTTTTCTACTTTGAAAATTCAAAAGAAATCGGCAGAGGAACATATGGATTAGTTTATAAAGCAGTTCCGAAAAAGCAAAATGGACAATTTCCTAACAAAGAATACGCATTAAAAATGATTGAGGGACAAGGATTTTCAATGTCTGCATGCAGAGAAATTGCTCTATTCAGAGAATTAAGgtaatttcaccgattaaataatattaatttaaaatgaagattgaattcgagattttcccctttttataatctaaaattcgaaaaaaaaactgaagtagatattcaaaa

**MAL450**: exon 2; SNP T>C (V40A)

ttggaaatttgcactataaataaatcagaaaatccctattgcagATTAATGATTGATGAAAACTTCAAAAAACAATTGGCACAGCGTCGAGAGAGAGTAGAAGACCTTTTCTACTTTGAAAATTCAAAAGAAATCGGCAGAGGAACATATGGATTAGCTTATAAAGCAGTTCCGAAAAAGCAAAATGGACAATTTCCTAACAAAGAATACGCATTAAAAATGATTGAGGGACAAGGATTTTCAATGTCTGCATGCAGAGAAATTGCTCTATTCAGAGAATTAAGgtaatttcaccgattaaataatattaatttaaaatgaagattgaattcgagattttcccctttttataatctaaaattcgaaaaaaaaactgaagtagatattcaaaa

**JU3643**[*cdk-8(mf167)*] and **JU3644** *cdk-8(mf168)*]: exon 2; SNP T>C (V40A); Synonymous SNPs

ttggaaatttgcactataaataaatcagaaaatccctattgcagATTAATGATTGATGAAAACTTCAAAAAACAATTGGCACAGCGTCGAGAGAGAGTAGAAGACCTTTTCTACTTTGAAAATTCAAAAGAGATTGGAAGGGGTACCTACGGTTTGGCTTATAAAGCAGTTCCGAAAAAGCAAAATGGACAATTTCCTAACAAAGAATACGCATTAAAAATGATTGAGGGACAAGGATTTTCAATGTCTGCATGCAGAGAAATTGCTCTATTCAGAGAATTAAGgtaatttcaccgattaaataatattaatttaaaatgaagattgaattcgagattttcccctttttataatctaaaattcgaaaaaaaaactgaagtagatattcaaaa

**JU3645**[*cdk-8(mf169)*] and **JU3646**[*cdk-8(mf170)*]: exon 2; PB306 SNP; Synonymous SNPs

ttggaaatttgcactataaataaatcagaaaatccctattgcagATTAATGATTGATGAAAACTTCAAAAAACAATTGGCACAGCGTCGAGAGAGAGTAGAAGACCTTTTCTACTTTGAAAATTCAAAAGAGATTGGAAGGGGTACCTACGGTTTGGTTTATAAAGCAGTTCCGAAAAAGCAAAATGGACAATTTCCTAACAAAGAATACGCATTAAAAATGATTGAGGGACAAGGATTTTCAATGTCTGCATGCAGAGAAATTGCTCTATTCAGAGAATTAAGgtaatttcaccgattaaataatattaatttaaaatgaagattgaattcgagattttcccctttttataatctaaaattcgaaaaaaaaactgaagtagatattcaaaa

***Cel-R09F10.3***

**PB306**: exon 4; MAL488 deletion

gtaagccgttatagctttgaaactaattagacgtattcaatttattttttagCAAACATTTTTTTATCTGTCGGAATTGCTACGTTGTGGGTCGACTCGTAAATACAGCAACCTTCCGGAACAGAGACTAGTTCCACCGGCAATGTTCCATTCGCAGGCAACAAATTCGCATGCAGCCTTTGAGCATGCATTCAATGGATTGAGAAAGGAAATTGCCAATAAGTCACTTGGAATCTATCCGAAGGTCCTGCAGCGAACCACTTGCAGTCTTATCATTGAAgtaagttgttgacttttttgattgaaaatatttttttaaaactcattttttccag

**MAL488**: exon 4; deletion breakpoint

gtaagccgttatagctttgaaactaattagacgtattcaatttattttttagCAAACATTTTTTTATCTGTCGGAATTGCTACGTTGTGGGTCGACTCGTAAATACAGAGACTAGTTCCACCGGCAATGTTCCATTCGCAGGCAACAAATTCGCATGCAGCCTTTGAGCATGCATTCAATGGATTGAGAAAGGAAATTGCCAATAAGTCACTTGGAATCTATCCGAAGGTCCTGCAGCGAACCACTTGCAGTCTTATCATTGAAgtaagttgttgacttttttgattgaaaatatttttttaaaactcattttttccag

**JU3647**[*R09F10.3(mf171)*] and **JU3648**[*R09F10.3(mf172)*]: exon 4; deletion breakpoint

gtaagccgttatagctttgaaactaattagacgtattcaatttattttttagCAAACATTTTTTTATCTGTCGGAATTGCTACGTTGTGGGTCGACTCGTAAATACAGAGACTAGTTCCACCGGCAATGTTCCATTCGCAGGCAACAAATTCGCATGCAGCCTTTGAGCATGCATTCAATGGATTGAGAAAGGAAATTGCCAATAAGTCACTTGGAATCTATCCGAAGGTCCTGCAGCGAACCACTTGCAGTCTTATCATTGAAgtaagttgttgacttttttgattgaaaatatttttttaaaactcattttttccag

***C. elegans* 54kb Deletion in PB306**

**MAL418** 54kb Deletion; Y75B8A.8 exon 1 Y75B8A.14 3’UTR deletion breakpoint ; inserted sequence

cacacacccaccacccatctaaacgtacagaaaccaacaaaaaaaaacagtgattagaaaaATGCAAAATTTCGGTGGTCCCGGAGGTCCGATGTATGGAGGAGCTGGTGGTGGTGGTGGAGGACCACCACGGGGTCCACCACAGCAACCGCCTCAACCACAGGGTGGTGGTGTTGCAGTGCCGTTTCCGATTCCGCAGGGTATCACTCAGGCTCATTATCATCAGTATAAGgtatatagaaaaacttttaaaaaatccggcgaaaaaataaaattcccaccaaaaagaactgcgaccaatcagcgattcgctccgcccactttgcaaccaatcagatggagtgggcggagtttgaagccgagcaaaacccaattttttttaaaccaaaaaatgaaaaatttcgtagtttcaaactaaattttcatttttgaaaaaaaaaatcattttaaaactattttttaattttcgaaaaatcgaaaaaaaaagtcttaaaatttctaaaaattccaaatttttcggaattttttcacttcaaagctgatcatccttcaggtgacgaaattgcctctaaataataaaaaatcccgaaaacttctaaaaattttgaatttgcgacaaaaattccacaaaatttgaaatttctcgatattcacaattcaaataactaaaaaaaaatatcTGATTCTGATTTTTTttctgattttttttcaaattttacactgaaaattagcaaaatatcgactttttataattttttagggcaaaattcgatgtatttatggcatattataatgaattatcgttttctcatgtttttttgcataaaaaatcaccaaaaataggccaaaaactgcaaaatcatgcgaaaaaaaattccagttagctaaaattctggtttttggagcattttttctgattttttttttcaaattttgcactgaaaattgacaatatatattgtttcaggtgaaaaagggacatttttaattttaaatgcaaaaaaaaattctgaaaaatcaaagattttcggctaaaaactgcaaaaccatgcgaaaaaatatatccgcacttgtccacgaggagtacaaaattcttcgtaaatcgacatgggaaaaaccgtgccgcgccgaagattttgcaatcttgcggcgcggtttttatgcatgtcgatttacggagaatcctgtactacacgtggacaagtctgagctatttttatattttttttgcattttttgtagctaaaattctggtttttggagcattttttaaatttttttcaaattttacactgaaaattgctaatcgtttttaatatatcgtttttaatctaaaatcaataatttcgttgttttttttttgaaaaaaatgaaaattttcgttttttccgatttttctggttttttggaagaaaattgtgtgttttcttatgtttttcttcacaaaaaaatcagcaaaaatttcggcaaaattgtccacgtgtagtacgaaatcctccgtaaatcgacatgggaaaaagcctgaaaaatgcctgaaaaaattcaatttttagcaggaattaaatggattttacgaaccaacaaattatatagaatcggggaaaaaaagtgtttaacaaattttaatcaacggggaagaaaaggcagaataaaacaaaaaaaagtaagtagtaacaaatcaacgaaaaaaaatcatttttcagtgcgaaaatttttccaaatttccgacca

**JU3357**[Y75B8A.8*(mf139)*]: exon 3; deletion breakpoint

tttcttttccaaaagattaaaatgaaaaaaataaccaaaaatccaaaaaaatttgtatttcagAATCCCACGGCCCGATTCCGGTCTCGAATCCGTCGTCTGCTCAGCAAATGCAATACTATCAGCAGCAGCAGCAGCAGCAGCTCCAACTTCAACAAAATCAGCAAATGCAAATGCAGCAGCAACAAATGCAGCAACATCAGCAGCAGCACCCGGGAATGGGTGGTCCAGAGATGGGTGGTCATATGCATGCTCAGCAGGGAGCTCCAGGCTCTCAGGCAGCACCAGTTCAGCAGCAGCAACAAATTCAGCAGCAGGCGCCTCCTCAAGCTCCAGCTGAAAAGACGGAAGATATGAAGTACAAGGAGCTGCTGAAAGAGATGAAGCTTCAGTACTTGGAGGCTCTGCAAGGAATGCAACGGCGGCAGGCTCAGCAGAAAGGTCTCGTGCAGATGGTGAATATACTTGAAGGCGATCGAATCGTCAGCTACGATCATCTGCTGAGTCTGAAGACCCCTCTGCATCGCCTGATGACCAGGGATTGCCCGACGTTCCCACTGATGGAAGAGATCCGGAAGGTTGTGTTCAAGAAGAAGGAGGATCGAGAGAAGGCGATGTTGATGAGCTTTGTTGGGGAGAAGGATAATCTTAGAAAGTGCGGATAAACGACTCTCCGAGCAGTCGAAGGATGATGATCCGATGGGGGTGAAGCCGTGGCGATCGGTGAAGCATTTGACGATTCGGGTGCCGGATTTTGTGAGGAATCTCACTGGGAATGAGGATAGGAAGGCGTTTCTGAAACGGCCACGAGCGGTGTCCGCGGAGGAAGATGAAGCTGTGGTGGGGGCGAAGAAGGTGAAGGAAGAGGAGCAGGATGATACGTCGAGTCAAGCATCCGAAGATGAGGATATTAGTCTGATACAGTCGGAGTTTGTGgtgtg

**JU3259**[*gly-11(mf119)*]: exon 2; inserted sequence

acctacttttgaattttcaatgttcaaattattccagAACATCGCCGCTCCACCAAAAATCATTCACAAAATCATTCACAACATTCCCGGATCGTTCAAAAGAAATTGAGATTGACACAGATTTGCTGGGGAAAATAAATGGAAAAGCCGAAGATGATCTACAAGTTGAAGGTTATAAAAAGTATCAGTTTAACGGATTACTAAGTGATCGAATTGGATCAAGAAGGAAGATCAAGGACTCTAGAAATGCCAGATGCTCTTCTCTAACATATTCCGACAGCTTACCAGCTGCCTCCATAGTTGTTTGTTATTTCAATGAATCACCTTCCGTGCTAATCCGAATGGTGAATTCAATTTTCGATCGTACAAAACCAGAACATCTCCATGAAATTCTACTTGTGGATGATTCAAGTGAATGGTCGAATGCAACTGATGAAGCTATCAAGTACAGAGAAAAACATATTATTCAATGGGAAAAAGTAAAGTTTTTGAAGACTGACAAGAATGAGGGATTGATTAGAGCAAAGATTTTTGGAGCAAGAAGGGCTAATGGAGAAGTTTTGgtaagttactattctctaatgtgaagagtagggcgataggcagg

**JU3260**[*gly-11(mf120)*]: exon 2; deletion breakpoint

acctacttttgaattttcaatgttcaaattattccagAACATCGCCGCTCCACCAAAATCATTCACAACATTCCCGGATCGTTCAAAAGAAATTGAGATTGACACAGATTTGCTGGGGAAAATAAATGGAAAAGCCGAAGATGATCTACAAGTTGAAGGTTATAAAAAGTATCAGTTTAACGGATTACTAAGTGATCGAATTGGATCAAGAAGGAAGATCAAGGACTCTAGAAATGCCAGATGCTCTTCTCTAACATATTCCGACAGCTTACCAGCTGCCTCCATAGTTGTTTGTTATTTCAATGAATCACCTTCCGTGCTAATCCGAATGGTGAATTCAATTTTCGATCGTACAAAACCAGAACATCTCCATGAAATTCTACTTGTGGATGATTCAAGTGAATGGTCGAATGCAACTGATGAAGCTATCAAGTACAGAGAAAAACATATTATTCAATGGGAAAAAGTAAAGTTTTTGAAGACTGACAAGAATGAGGGATTGATTAGAGCAAAGATTTTTGGAGCAAGAAGGGCTAATGGAGAAGTTTTGgtaagttactattctctaatgtgaagagtagggcgataggcagg

**JU3354**[Y75B8A.10*(mf135)*]: no PCR band in exon 5 with:

A10_PCR_5F: ACAGTTCGATGAAAGAGACGC

A10_PCR_4R: TTAGCTAACCTCCTCCACCG

A10_SANGER_F: CCCCTGGACACTATGTAAAGC

A10_SANGER_R: GCGGCTCGATTGTAAAACTAAA

**JU3355**[Y75B8A.44*(mf138)*]: exon 1; deletion breakpoint

ATGGGCGGCACCATCCTCTCAGCCGCAAGCCTTCTCATTTTGTCTCAGAAAACGGAGGAAGAAATGCGTGGAATTGTGATTCTTCTGCTCATTGTCGTCAGCCTACTGCTCATTTCGACTGTTCACTCGCATCCGTTCTTCATCGCACTCACAAGATTACAG

**JU3257**[*osm-12(mf117)*]: exon 4; inserted sequence

ccagAGTTATCTTTGCACGGAAGACATTCATGATGTTGTAAGCCTTGTATTCGAAGAAGCATGGGGGAGTCGAGAATACACTTCAATTCTCGCATGTGGTAACTCAACTCTTCAAATCATTGAAGGCAACAACTTCGCCTATGATGTCCGACTCGATAGTGTTCCATTCACAGTTTCCCTTTTTATGGGCGATGGTGGTCATACGAAGCTTTTGGTTCTTTACGGAACAAAAACTGGCAGGCTGGGACTTGTTAGTGTACCTCAAGTTAGTGTACCTTTGTTGAAGTTGCAAAATTCTTATGAAAAAAAAGGTGTTTTCAGTCTTGTGACGAGTCGATAACTGGAATATCGTGCGGTCATGTATCGGTGGAAAGATCCTATGGAAATTGATAGTTGCAAAATTCTTATGAAAAAAAAGGTGTTTTCAGTCTTGTGACGAGTCGATAACTGGAATATCGTGCGGTCATGTATCGGTGGAAAGATCCTATGGGAAATTGATACAACGTCGGGAGCTTGTGTCACAACAATTGTGTGCTTCAATGTGACTGGCGGACAATTTCCGGATATTATTGTGGGAAAAGAAGATGGATTGATTGAGATTTACGTTATTGATGAAACTGATCATGCACATTTATTCGGAACTTTTgt

**JU3261**[Y75B8A.13*(mf121)*]: exon 1; deletion breakpoint

agcacaaaaATGCCAGCCACTGCATCCTATTTCAAAGAAATTATCGATGATTATATTTTAAAATCGAAAATTGCTTATGATTTTTACAAAAAATCACAAGTTCTGATAACTTGCAAGgtaattttcaaaaaa

***Cbr-sfrp-1***

**HK104**: exon 2; MAL296 SNP A>C (N59H)

gtaaggaggtttatatcgaaaaactccatagatattttttgtattcggaacccatttcttttttctagTATACACAAATGCGTCTTCCAAATATCCTGGAGCATGAAACAGTCTCCGAAGCAATTCACGCATCAAAAGATTGGGAAAGTCTTCTTCGTTTAAATTGTCATCCAGATACACAGgttcgtgatggt

**MAL296**: exon 2; SNP A>C (N59H)

gtaaggaggtttatatcgaaaaactccatagatattttttgtattcggaacccatttcttttttctagTATACACAAATGCGTCTTCCACATATCCTGGAGCATGAAACAGTCTCCGAAGCAATTCACGCATCAAAAGATTGGGAAAGTCTTCTTCGTTTAAATTGTCATCCAGATACACAGgttcgtgatggt

**JU3707**[*Cbr-sfrp-1(mf177)*]: exon 2; SNP A>C (N59H); Synonymous SNPs

gtaaggaggtttatatcgaaaaactccatagatattttttgtattcggaacccatttcttttttctagTATACACAAATGCGTCTTCCACATATTCTCGAACACGAGACCGTTTCTGAAGCAATTCACGCATCAAAAGATTGGGAAAGTCTTCTTCGTTTAAATTGTCATCCAGATACACAGgttcgtgatggt

**JU3708**[*Cbr-sfrp-1(mf178)*]: exon 2; HK104 SNP; Synonymous SNPs

gtaaggaggtttatatcgaaaaactccatagatattttttgtattcggaacccatttcttttttctagTATACACAAATGCGTCTTCCAAATATTCTCGAACACGAGACCGTTTCTGAAGCAATTCACGCATCAAAAGATTGGGAAAGTCTTCTTCGTTTAAATTGTCATCCAGATACACAGgttcgtgatggt
