# Supplementary material for: A broad mutational target explains a fast rate of phenotypic evolution
Source: eLife. 2020 Aug 27;9:e54928. doi: 10.7554/eLife.54928 (PMC7556874; doi:10.7554/eLife.54928)
Supplement: Transparent reporting form [file elife-54928-transrepform.docx]

***eLife’s* transparent reporting form**

We encourage authors to provide detailed information *within their submission* to facilitate the interpretation and replication of experiments. Authors can upload supporting documentation to indicate the use of appropriate reporting guidelines for health-related research (see [EQUATOR Network](http://www.equator-network.org/%20%20\t%20_blank)), life science research (see the [BioSharing Information Resource](https://biosharing.org/)), or the [ARRIVE guidelines](http://www.plosbiology.org/article/info:doi/10.1371/journal.pbio.1000412) for reporting work involving animal research. Where applicable, authors should refer to any relevant reporting standards documents in this form.

If you have any questions, please consult our Journal Policies and/or contact us: [editorial@elifesciences.org](mailto:editorial@elifesciences.org).

**Sample-size estimation**

- You should state whether an appropriate sample size was computed when the study was being designed
- You should state the statistical method of sample size computation and any required assumptions
- If no explicit power analysis was used, you should describe how you decided what sample (replicate) size (number) to use

Please outline where this information can be found within the submission (e.g., sections or figure legends), or explain why this information doesn’t apply to your submission:

**Replicates**

In the present work, the traits we use are frequencies. We did not perform an explicit power analysis prior to experiments. As indicated in Methods, Section 'Scoring the cell fates of P(3-8).p, the number of animals scored is a compromise between the number of assayed lines and the number of biological replicates. We generally scored 50 animals per strain at each experimental replicate (min=10, max=116, average=55): it provides a reasonable confidence interval while keeping it compatible with scoring multiple lines in the same experimental block.

**See Suppl File 1**: the last column "n" indicate the number of animals assayed for each experimental block, providing an estimate of P3.p division frequency.

- You should report how often each experiment was performed
- You should include a definition of biological versus technical replication
- The data obtained should be provided and sufficient information should be provided to indicate the number of independent biological and/or technical replicates
- If you encountered any outliers, you should describe how these were handled
- Criteria for exclusion/inclusion of data should be clearly stated
- High-throughput sequence data should be uploaded before submission, with a private link for reviewers provided (these are available from both GEO and ArrayExpress)

Please outline where this information can be found within the submission (e.g., sections or figure legends), or explain why this information doesn’t apply to your submission:

We report the data structure for each experiment. Most strains were scored multiple times in different "experimental blocks" (different days, different animal cultures) to ensure biological consistency of the estimate of P3.p division frequency (up to 32 replicates for progenitor line PB306). RILs and back-crossed lines were generally scored once with 50 animals, as this was sufficient to classify the lines as PL-like or MA line-like (Fig. 3).

We include a definition of biological replicate in Methods, Section 'Scoring the cell fates of P(3-8).p'. With a binary trait, each animal is observed once: there is no 'technical replicate' and there are no outlier individuals. We did not see outlier lines, for example in the backcrossing experiments. We did not exclude data.

Suppl File 1 indicates the number of blocks for each strain.

The high-throughout data are already publically available (accession numbers in Suppl File 2).

**Statistical reporting**

- Statistical analysis methods should be described and justified
- Raw data should be presented in figures whenever informative to do so (typically when N per group is less than 10)
- For each experiment, you should identify the statistical tests used, exact values of N, definitions of center, methods of multiple test correction, and dispersion and precision measures (e.g., mean, median, SD, SEM, confidence intervals; and, for the major substantive results, a measure of effect size (e.g., Pearson's r, Cohen's d)
- Report exact p-values wherever possible alongside the summary statistics and 95% confidence intervals. These should be reported for all key questions and not only when the p-value is less than 0.05.

Please outline where this information can be found within the submission (e.g., sections or figure legends), or explain why this information doesn’t apply to your submission:

The statistical tests are reported in Figure legends. Raw data are in supplemental tables (Suppl File 1 for P3.p scores with N and replicate structure). Note that we do not have data reports regarding each individual as they are scored visually with a microscope. Statistical analysis methods are described in the section Material and methods/Statistical Analysis.

Besides Suppl File 1, N per block/number of blocks are visible on all figures, either given as number in a table (Fig. 2b) or as point size/number of points in plots (Fig. 2b, Fig. 3, Fig. 4). We also provide 95% confidence intervals for the estimate of P3.p division frequencies as error bars in barplots of Fig. 2b, Fig. 3, Fig. 4 b-f. We report groups of strains with no statistical difference (e.g. Fig.4).

(For large datasets, or papers with a very large number of statistical tests, you may upload a single table file with tests, Ns, etc., with reference to sections in the manuscript.)

**Group allocation**

- Indicate how samples were allocated into experimental groups (in the case of clinical studies, please specify allocation to treatment method); if randomization was used, please also state if restricted randomization was applied
- Indicate if masking was used during group allocation, data collection and/or data analysis

Please outline where this information can be found within the submission (e.g., sections or figure legends), or explain why this information doesn’t apply to your submission:

Not applicable.

No masking was used (indicated in Methods, Section 'Scoring the cell fates of P(3-8).p)'.

**Additional data files (“source data”)**

- We encourage you to upload relevant additional data files, such as numerical data that are represented as a graph in a figure, or as a summary table
- Where provided, these should be in the most useful format, and they can be uploaded as “Source data” files linked to a main figure or table
- Include model definition files including the full list of parameters used
- Include code used for data analysis (e.g., R, MatLab)
- Avoid stating that data files are “available upon request”

Please indicate the figures or tables for which source data files have been provided:

Suppl File 1 provides P3.p scoring data (Fig. 2, Fig. 3, Fig. 4).

Suppl File 2 provides the accession numbers for genomic data.

Suppl File 3 provides data for analysis of mutations found in our set of MA lines (Fig. 5).
